# A Combined Neuroanatomy, Ex Vivo Imaging, and Immunohistochemistry Defined MRI Mask for the Human Paraventricular Nucleus of the Thalamus

**DOI:** 10.1002/hbm.70366

**Published:** 2025-09-25

**Authors:** Madison R. Tetzlaff, Bianca T. Leonard, Michael A. Yassa, Tallie Z. Baram, Jerod M. Rasmussen

**Affiliations:** ^1^ Department of Anatomy and Neurobiology University of California Irvine California USA; ^2^ Department of Neurobiology and Behavior University of California Irvine California USA; ^3^ Center for the Neurobiology of Learning and Memory University of California Irvine California USA; ^4^ Department of Pediatrics University of California Irvine California USA; ^5^ Department of Biomdical Engineering University of California Irvine California USA

**Keywords:** fMRI, functional connectivity, human, magnetic resonance imaging, midline thalamus, paraventricular nucleus of the thalamus, post‐mortem

## Abstract

The paraventricular nucleus of the thalamus (PVT) is an evolutionarily conserved midline thalamic structure known to contribute to arousal, interoceptive states, and motivated behaviors. Yet, a consensus anatomical definition of the human PVT across tissue‐based and MRI‐based approaches remains elusive, thereby limiting reliable translation between its cellular characteristics and in vivo functional connectivity. To address this challenge, we describe a histologically informed PVT segmentation compatible with standard 3 T MR imaging pipelines. We performed postmortem anatomical MRI scans on an intact whole brain and an excised thalamic block, manually segmented the PVT at high resolution using ex vivo calretinin staining and neuroanatomical landmarks, registered the resulting image‐label pair to a commonly used MRI template space (Montreal Neurological Institute's MNI152), and performed a comparative reanalysis using this newly defined mask. This tissue‐grounded PVT mask largely overlaps spatially with existing MRI‐based PVT masks, with the exception of additional voxels posteriorly. Importantly, the functional connectivity patterns of this tissue‐grounded mask are highly consistent with those previously reported. Collectively, this multimodal definition of the human PVT balances tissue‐based ground truth with in vivo MRI features, providing a valuable resource for advancing translation between cellular level features identified by histology and in vivo functional connectivity at the meso/macro scale in the understudied human PVT.

## Graphical abstract

Existing definitions of the human paraventricular nucleus of the thalamus (PVT) differ between histological and MRI atlases; this study addresses this conflict by leveraging MRI and histology in the same post‐mortem brain. Despite anatomical differences, functional connectivity of the histologically defined PVT and previous MRI atlases varies marginally.

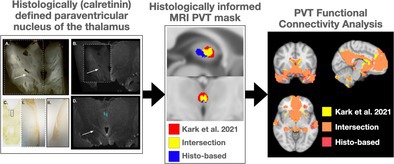

**Summary**

- Addresses conflict between histological and MRI‐based borders of the human PVT.
- Histologically defined PVT boundaries indicate additional posterior tail to the human PVT as compared to existing human MRI atlases.
- Provided MRI mask demonstrates functional connectivity patterns consistent with prior human and rodent studies.

## 1 Introduction

The paraventricular nucleus of the thalamus (PVT) is a midline thalamic nucleus that is conserved across species. It has been extensively studied in animal models ranging from lizards to rodents, underscoring its fundamental role in a wide array of behaviors (Bhatnagar et al. 2002; Bhatnagar and Kirouac 2021; Fenoglio et al. 2006; Gao et al. 2023; Gao et al. 2020; Hain et al. 2022; Heredia et al. 2002; Kooiker et al. 2021). Specifically, the PVT has been linked to arousal, hunger, salience detection, and the processing of internal states (Ye et al. 2022; Millan et al. 2017; Labouèbe et al. 2016; Engelke et al. 2021; Do‐Monte et al. 2015; Kooiker et al. 2023; Choi et al. 2019; Choi and McNally 2017; McNally 2021; Beas et al. 2024). The PVT is thought to play a critical role in regulating motivated behaviors, particularly by resolving conflicts between competing motivational drives. Despite its demonstrated importance, the human PVT remains relatively understudied, with a paucity of information regarding its cellular and chemical composition as well as its in vivo connectivity and functions.

Disagreement on the anatomical boundaries of the human PVT is a major barrier to its reliable study. Limited work directly examining the chemical and cellular composition of the human PVT includes (Uroz et al. 2004; Schulmann et al. 2024 preprint) neither of which describes clear boundaries or cell type variations across the PVT. In contrast, the rodent PVT has been extensively examined by immunohistochemistry, spatial transcriptomics, and sequencing methods, and describes an important distinction between the anterior and posterior PVT (Gao et al. 2023; Shima et al. 2023; Beas et al. 2024; Kooiker et al. 2021; Choi et al. 2019). The issue of anatomical boundaries in the human thalamus is not unique to the PVT; ambiguities in terminology and boundary definitions exist for all thalamic nuclei (Mai and Majtanik 2018). Several atlases have been created in an attempt to unify these boundaries, though some group the PVT under the broader definition of “midline thalamus”, and others do not delineate the PVT at all, underscoring the extent to which its boundaries remain unsettled (Erzurumlu et al. 2024; Saranathan et al. 2021; Reeders et al. 2023; Pfefferbaum et al. 2023).

Recent in vivo human studies have relied on a putative three‐dimensional thalamic atlas built from the Morel stereotactic atlas (Morel 2007; Krauth et al. 2010; Jakab et al. 2012; Kark et al. 2021), which includes PVT boundaries from eight post‐mortem human brains. Using this atlas, work by several groups, including our own, suggests that human and rodent PVT networks share analogous target nodes, including the nucleus accumbens, amygdala, hypothalamus, periaqueductal grey, and hippocampus (Li and Kirouac 2012; Otis et al. 2019; Kark et al. 2021; Kooiker et al. 2021; McGinty and Otis 2020). Moreover, these connections appear behaviorally relevant, as they have been linked with prolonged maternal grief after loss of a child (Kark et al. 2022), anhedonia in adult males (Leonard et al. 2024), and craving in cocaine use disorder (Engeli et al. 2023). While these recent efforts with fMRI have advanced understanding of human PVT function, clear and mechanistic interpretation of these results is limited by the lack of clear, universally accepted neuroanatomical boundaries.

The Krauth MRI atlas (constructed using the Morel stereotactic atlas) represents the current State of the Science for fMRI of the human thalamus and serves as the foundation for most human PVT research, but the average‐based delineation of nuclei may blur individual anatomical boundaries. For example, marked differences in PVT boundaries emerge when comparing the Morel‐defined PVT borders with individual subject histology, such as that in the Allen Brain Atlas (Ding et al. 2016), the Paxinos atlas of the human brain (Mai et al. 2004), and the Nextbrain atlas (Casamitjana et al. 2024). The Morel and Krauth atlases appear to omit the posterior extent of the human PVT, but it is unclear if this represents an important or detectable variation in human anatomy and function. Thus, there is a pressing need for a histologically validated definition of the PVT to support ongoing in vivo human neuroimaging studies and interpretation of these studies in the context of cellular characteristics.

Here, we address anatomical inconsistencies in PVT delineation by constructing a “ground‐truth” MRI‐compatible three‐dimensional mask based on histological markers from a single post‐mortem human brain. We define PVT boundaries based on the positions of white matter tracts and the continuous, circumscribed expression of calretinin, an established marker of the PVT across multiple species including human, primate, cat, rat, mouse, and lizard (Fortin et al. 1996; Gao et al. 2023; Hua et al. 2018; Uroz et al. 2004; Winsky et al. 1992; Mátyás et al. 2018; Viena et al. 2021). This combined approach identifies a set of boundaries for the PVT common across postmortem histology, neuroanatomy, and MRI‐visible features. In addition, we reconcile discrepancies with the commonly used Morel atlas through a descriptive analysis of these boundaries and a reanalysis of MRI‐based in vivo functional connectivity data.

## 2 Methods

**FIGURE 1:**
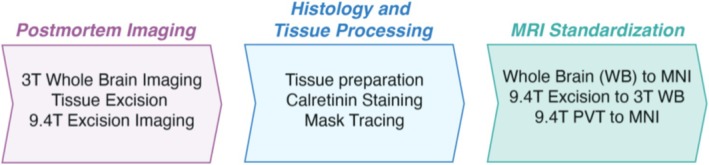
Overview of experimental framework. Postmortem MRI scans were acquired at two different resolutions, before and after the excision of the bilateral thalamic region (“thalamic block”). Histological processing followed, including calretinin staining for enhanced visualization of the PVT region. Guided by the histology results, a three‐dimensional PVT mask was segmented. Through linear and nonlinear transformations, this mask was rendered in standard MNI152 template space for functional connectivity analysis using fMRI.

The overall design and workflow of the experiments is shown in Figure 1. Briefly, the postmortem brain was scanned at 3 T, and then the thalamus and overlying ventricular system were dissected out to a block and re‐scanned at both 3 and 9.4 T. After completion of MRI scans, the thalamic block was further processed into sections, stained for calretinin, and the PVT was traced back onto the highest resolution MRI scan. Finally, the resulting PVT mask was transformed into MNI standard space, and the functional connectivity was assessed as compared to that of our previously published MRI mask (Kark et al. 2021). A more detailed description of these procedures is provided below.

### 2.1 Post‐Mortem MRI

A 67‐year‐old male with no known neuropsychiatric or neurodegenerative disease contributed his brain to the UCI Willed Body program and formed the basis of the procedures and analyses performed here. On receipt of the donor brain, we transferred the tissue from 10% formalin through several rinses of 1× phosphate buffer solution (PBS). Between steps and unless otherwise noted, the whole brain and subsequent dissections were stored in 1× PBS. A morphological assessment of the thalamus based on MRI was conducted to establish no major morphological differences relative to a representative cohort (see Figure S1).

Prior to histological processing, we acquired a series of high‐resolution MR images of the whole postmortem brain to facilitate the creation of a histologically grounded PVT mask in standard template space. Each acquisition was designed to optimize registration and anatomical fidelity across modalities. First, we obtained a standard and high‐resolution whole‐brain T1‐weighted image at 3 T to maximize intensity and contrast information for registration to the MNI template. The brain was then dissected to remove cortical structures, leaving the bilateral thalamus untouched and the ventricular system largely intact (Figure S2). The dissected thalamic block was re‐scanned at 3 T using identical sequences to the whole‐brain MRI to capture image details while accounting for deformations introduced by the dissection and facilitating registration of histological tracings to MNI space. Finally, the thalamic block was scanned at 9.4 T with ultra‐high resolution, ensuring precise anatomical correspondence with the histological images. Collectively, these imaging steps serve as intermediate reference points, maximizing information overlap and ensuring accurate registration between histology and the commonly used MNI152 MRI template. Specific acquisition details are provided below.

### 2.2 Whole Brain and Excised (“Thalamic Block”) 3 T MRI Acquisition

Postmortem MRI imaging was conducted on a 3 T Siemens MAGNETOM Prisma scanner. The imaging protocol included a high‐resolution structural MPRAGE sequence (0.8 mm isotropic resolution; 320 sagittal slices, field of view = 240 × 256 mm, flip angle = 4, TR/TE = 5000/3.2 ms, matrix size = 300 × 176, inversion pulse TI = 700 ms). Following whole‐brain imaging, the thalamic block, which contained the PVT and adjacent structures, was immersed in fluorinert and re‐scanned at 3 T using identical imaging parameters to maintain consistency while accounting for potential post‐processing deformations.

### 2.3 Excised 9.4 T MRI Acquisition

The block was then rescanned using a 9.4 Tesla (9.4 T) Bruker scanner to obtain ultra‐high resolution structural imaging. A spin echo sequence was used (0.6 mm isotropic resolution; 224 sagittal slices, field of view = 200 × 200 mm, TR/TE = 1100/3.2 ms, matrix size = 320 × 320).

### 2.4 Histology and Tissue Processing

Following the 9.4 T scan, the excised block was submerged in 15% sucrose in 0.1 M phosphate buffer followed by 30% sucrose in phosphate buffer, allowing the tissue to sink in each solution to ensure proper water removal and cryoprotection. The tissue was then trimmed and sectioned into 1.5 cm coronal slices for ease of handling, embedded in OCT (*Tissue‐Tek*, *#4583*) in flat trays (Figure S2), and snap‐frozen in a bath of isopentane chilled by dry ice. The frozen tissue was sectioned at 40 μm using a Leica CM1900 cryostat and stored in 0.1 M phosphate buffered saline (PBS) with 0.01% sodium azide at 4°C.

Due to prolonged fixation (> 18 months) in 10% formalin, formalin stripping was required to unmask the calretinin epitope. This was accomplished via incubations in 1 M EDTA (pH 8.0) and 1× Triton, heated to near boiling and maintained at this temperature via microwave bursts (~10 s each) for 20 min. Following formalin stripping, the specimen was rinsed in 1× PBS with Triton (PBST), treated with 30 μL/10 mL of 30% H_2_O_2_ for 30 min, then blocked for 1 h in 10% normal goat serum (NGS). Primary antibody incubation for 36–48 h at 4°C with polyclonal rabbit anti‐calretinin (1:250, *Invitrogen, #180211*) in 2% NGS in 1× PBST was followed by secondary incubation for 2 h at room temperature in biotinylated goat anti‐rabbit (*Invitrogen, #3182G*) in 1× PBST. After antibody incubations, tissue was incubated with ABC‐Kit (*Vector Labs, PK‐4000*) for 2–3 h per manufacturer instructions. Tissue was allowed to react with activated DAB (*Vector Labs, SK‐4100*) for 12–15 min before ceasing the reaction. When used, hematoxylin (*Abcam, AB220365*) was applied for 30 s before rinsing with 1× PBST. Microscopy images were taken with a Nikon Eclipse E4000 microscope with the use of the Nikon FS Elements acquisition software. Gross anatomical images were taken with an iPhone 13.

### 2.5 Alignment of the Tissue Specimen With Post‐Mortem MRI and Individual PVT Segmentation

**FIGURE 2:**
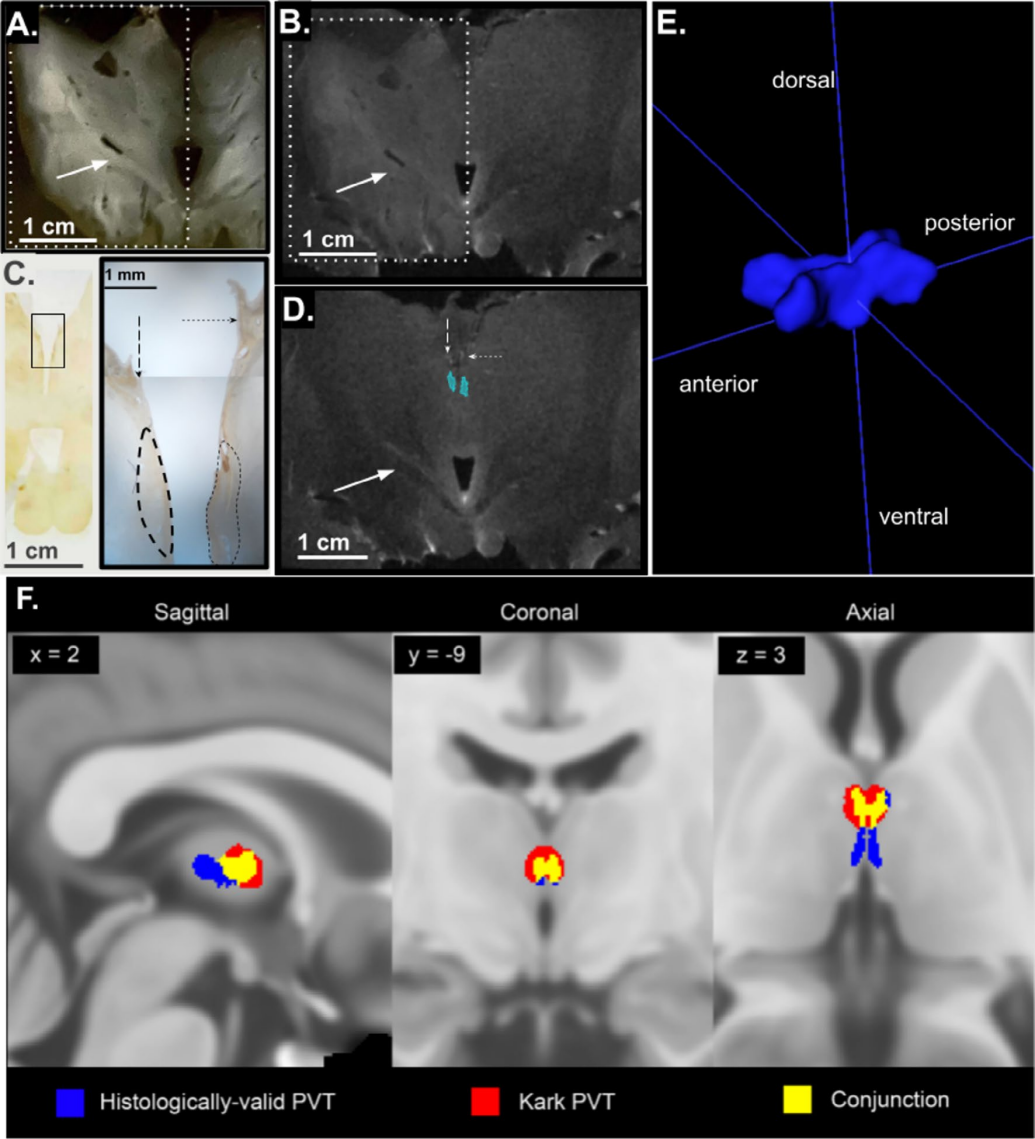
Alignment of histology to MRI produces posterior tail on PVT mask. Example alignment of tissue slice to MRI section, showing wet, unstained slice with distinguished white matter tracts (A) aligned to MRI slice in (B). White dotted border in (A) outlines part of the image which is made 50% opaque and overlaid on (B). Calretinin‐stained slide (C) neighboring (A) with inset to the right showing the bilateral calretinin‐positive region traced onto the MRI slice in blue in (D). Dotted arrows indicate the stria medullaris thalami. Solid arrow indicates common white matter tract in the same location across slices. (E) Three‐dimensional rendering of final PVT mask in MNI152 standard MRI space (volume = 1583 voxels (~198 mm^3^)). (F) Overlay of multiple PVT masks in MNI152 standard MRI space (red; Kark et al. 2021) and the newly generated PVT mask (blue) to visualize shared voxels from three viewpoints (yellow, Dice‐Sørenson coefficient (DSC) = 0.37).

Tracing of the PVT onto the 9.4 T MRI scan of the excised thalamic block was completed by one experimenter (MT) using direct microscopy observation (magnification 1×–20×) of one immuno‐stained tissue section per MRI slice and histologically and anatomically grounded features. Specifically, gross anatomical markers including the mammillary bodies, red nucleus, mammillothalamic tract, habenular commissure, other white matter tracts, and the ventricular contours were used as landmarks to align the immuno‐stained tissue section with a single MRI slice. Neighboring unstained tissue sections were used to verify alignment of the calretinin‐immunostained section to the MRI plane, as in Figure 2A–D. Regions determined to be PVT were manually segmented over coronal slices of the 9.4 T excised thalamic MRI image (0.2 mm isotropic) to generate a 3D seed region using 3D Slicer (v5.6.2). Regions of the midline thalamus were determined to be PVT by the following criteria: (1) circumscribed calretinin expression, (2) bordering the third ventricle, and (3) dorsal to any other circumscribed regions of calretinin positivity (i.e., nucleus reuniens). Every sequential MRI slab contained a PVT segmentation overlapping at least partially with the segmentation on neighboring sections, representing a single entity in the antero‐posterior extent.

**FIGURE 3:**
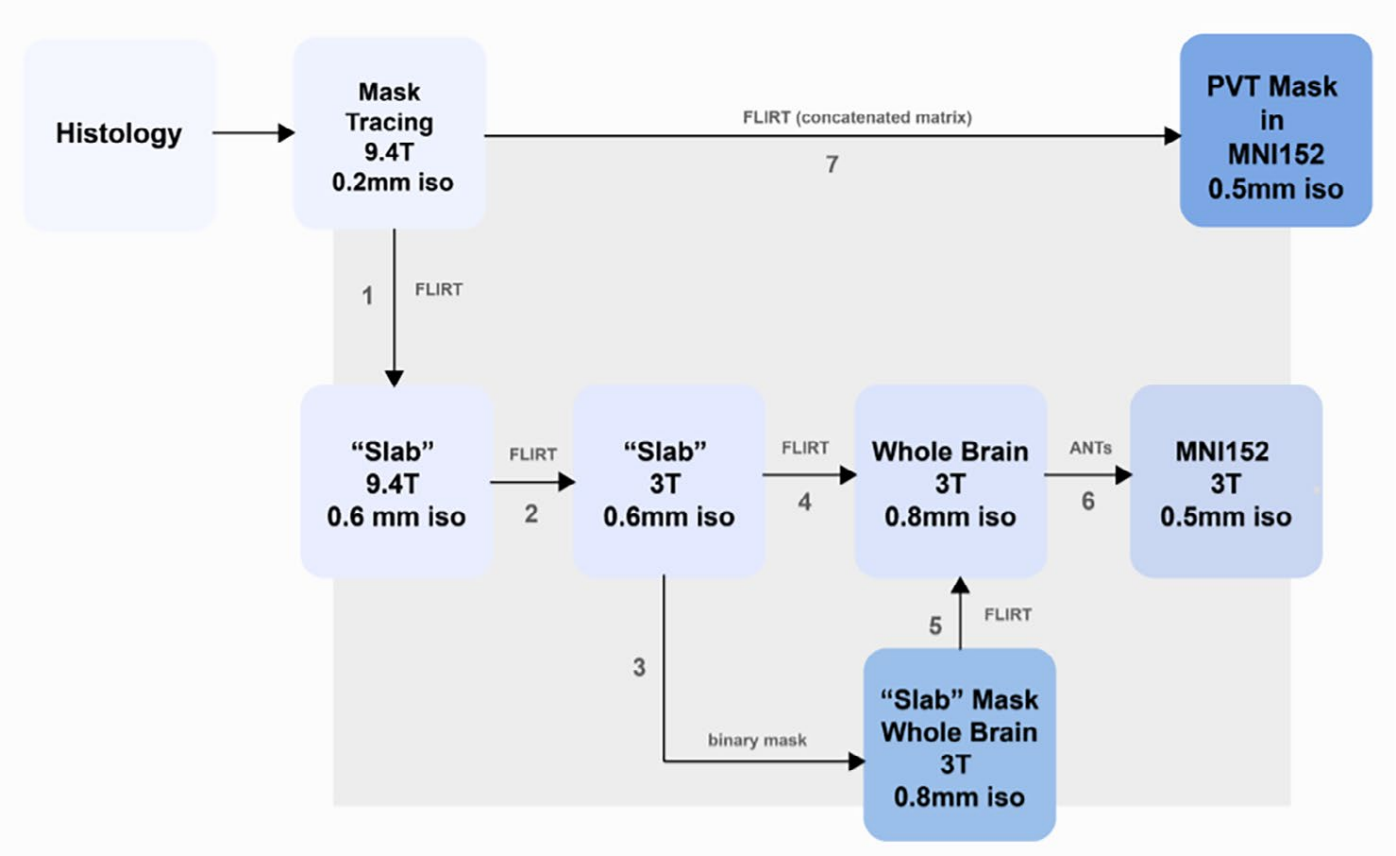
Registration pipeline transforms PVT mask from histological‐tracing space into MRI standard space (MNI152). To generate a PVT mask based on calretinin and histological information, several structural images were captured at differing resolutions of the whole postmortem brain and an excision of the thalamus and surrounding tissues (“block”) as described in the squares of Steps 2–5. The stepwise transformations applied to the images were concatenated into one matrix and applied to the PVT mask traced prior to Step 1 for the generation of the final PVT following Step 7.

The first step in generating a transformation matrix to place the PVT segmentation in standard space involved aligning the histological tracing on the 9.4 T image (0.2 mm resolution) to the 0.6 mm resolution image from the same scanner using linear transformation (Figure 3, Step 1). This was performed using FMRIB's Linear Image Registration Tool, FLIRT, which is part of the FMRIB Software Library (FSL, Jenkinson et al. 2012).

### 2.6 Alignment of 9.4 T Thalamic Block to 3 T Postmortem Whole Brain

To generate an artificial excision from the successfully registered 3 T whole‐brain scan, the 9.4 T image of the thalamic block was registered to the 3 T image of the same block (Figure 3, Step 2). A binary mask of the 3 T thalamic block was generated within the whole brain to support the registration of the 3 T thalamic block to the 3 T whole brain image (Figure 3, Steps 3, 4). Finally, the 3 T thalamic block and whole‐brain images were registered using the nonlinear transformation matrix generated in the next step, aligning the 3 T whole‐brain scan to MNI152 standard space (Figure 3, Step 5).

### 2.7 Alignment of 3 T Postmortem Whole Brain to MNI152 Standard

To facilitate the creation of an MRI atlas in standardized space, we performed brain extraction (Brain Extraction Tool, BET) and linear registration of the 3 T postmortem whole‐brain scan. A nonlinear transformation (12 DOF) was then applied using Advanced Normalization Tools (ANTs, Tustison et al. 2021) to align the scan with the MNI152 brain template at 0.5 mm resolution (Figure 3, Step 6).

### 2.8 Alignment of the Histology Informed PVT Segmentation to MNI152 Standard

The transformation matrices from the previous section were concatenated and applied to the histology‐informed PVT mask using FLIRT (Figure 3, Step 7). The resulting voxels were thresholded at 0.5, after which the mask was binarized. This final step completed the transformation of the PVT segmentation from the native 9.4 T space into an MNI152 standard space PVT mask with a 0.5 mm resolution (Figure S3). To assess consistency, a Dice‐Sørensen coefficient (DSC) was calculated between the previous and updated PVT masks.

### 2.9 Statistical Analysis of Variation in Masks and Functional Connectivity

A critical comparison between the histologically defined PVT mask and the commonly used Morel atlas PVT mask revealed notable differences in anatomical definition. To assess the functional implications of these differences, we compared functional connectivity measures derived from the new histologically defined PVT mask with those derived from the Morel atlas PVT mask. This re‐analysis was conducted using previously published PVT connectivity maps (Kark et al. 2021), ensuring that all methodological aspects remained identical, except for the mask used.

The imaging sample for this functional connectivity analysis was drawn from the Human Connectome Project (HCP) young adult cohort. It included 121 young healthy adults (ages 22–35, 73 female, and 48 male) who underwent a 7 T resting state functional magnetic resonance imaging (rsfMRI) session. This sample comprised all remaining participants after exclusions for poor image quality, excessive motion in the scanner, or anatomical, behavioral, or neurological criteria flagged by the HCP protocol.

Full details of the image processing and functional connectivity analyses are available in our group's previous study (Kark et al. 2021). Briefly, we used the “minimally preprocessed” datasets provided by the HCP 1200 Release (HCP filename: rfMRI*hp2000_clean.nii.gz, *n* = 135) (Glasser et al. 2013). These datasets included structural and functional images that had undergone preprocessing, including independent components analysis for artifact removal of noise components from fMRI data (ICA‐FIX) (Salimi‐Khorshidi et al. 2014; Griffanti et al. 2014). Consistent with Kark et al., both structural and functional datasets were further processed using the CONN Toolbox for functional connectivity analyses (Smith and Nichols 2009; Whitfield‐Gabrieli and Nieto‐Castanon 2012). Within the CONN toolbox, data were segmented, smoothed, denoised, and processed for further artifact detection via Artifact Detection Tools (ART), where conservative motion censoring thresholds were applied, and 14 of the 135 participants were dropped for having fewer valid scans than the rest of the group (as defined by the 1st Q—1.5 IQR).

Functional connectivity seed‐to‐voxel maps were then generated using the new PVT segmentation. Connectivity was calculated by calculating semipartial correlations between the average BOLD signal of the PVT mask voxels and all other voxels in the brain. The PVT mask was subtracted from a whole‐thalamus mask (Jakab et al. 2012), and the signal from the remaining thalamic voxels was added as a regressor to the calculation of functional connectivity for the PVT. Given the small volume of the PVT mask, this was done to reduce signal from overlapping voxels in the PVT functional connectivity calculation. To determine significant connectivity patterns, we applied a threshold‐free cluster enhancement (TFCE) method with family‐wise error correction (pTFCE‐FWE < 0.05) with 1000 permutations. The degree of overlap between spatial maps was quantified using DSC. Coefficient of determination (*R* ^2^) values were calculated from *T*‐statistic maps of the functional connectivity measures to further quantify the similarity between each seed‐to‐voxel functional connectivity map.

## 3 Results

### 3.1 Tissue‐Based Definition of the Human PVT

**FIGURE 4:**
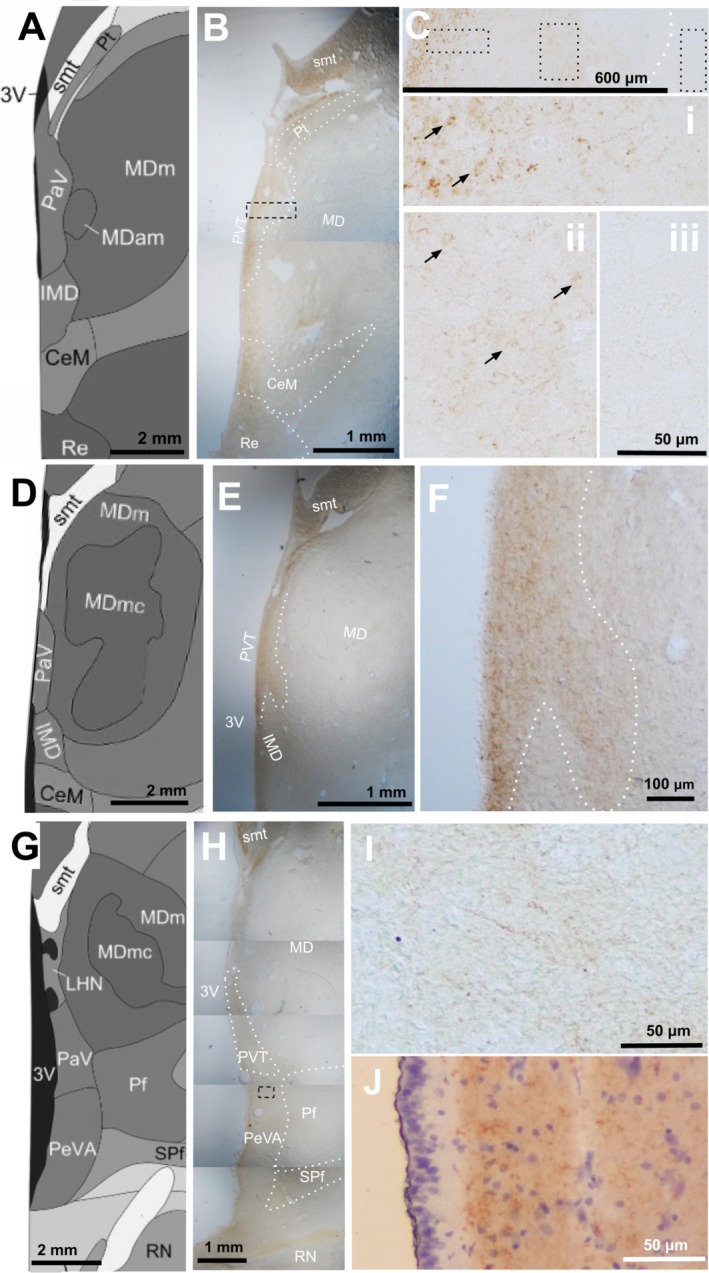
Calretinin delineates the paraventricular nucleus of the thalamus from neighboring nuclei in the coronal plane. Representative images from Allen Human Brain Reference Altas (A, D, G; *PaV = PVT*) and corresponding brightfield images of the anterior (B), middle (E) and posterior (H) human midline thalamus stained for calretinin, with the PVT outlined along the borders used to create the MRI mask. The dotted box on (B) indicates the higher magnification area shown in (C). From left to right, the dotted boxes in (C) correspond to the panels (i), (ii), and (iii), showing calretinin stained higher‐magnification images within and past the drawn boundary of the PVT at the same magnification. Arrows indicate cell bodies. At the mid‐PVT, there is a narrowing of the calretinin stain along the ventricle (F). Postero‐ventrally in the PeVA, the calretinin stain changes, detecting fibers with few to no cell bodies ((I), from location indicated by dotted box in (H)). The calretinin stain shows specificity of calretinin labeling away from the ependymal layer (J). Brown = Calretinin DAB+, purple = hematoxylin. 3 V, third ventricle; CeM, centromedial nucleus; IMD, intermediodorsal nucleus; MD, mediodorsal nucleus; MDm, mediodorsal nucleus, medial part; MDmc, mediodorsal nucleus, magnocellular part; PeVA, periventricular area of the thalamus; PF, parafascicular nucleus; PT, paratenial nucleus; PVT, paraventricular nucleus of the thalamus; RN, red nucleus; smt, stria medullaris thalami; SPf, sub‐parafascicular nucleus.

Histologically, the PVT was defined as a calretinin‐positive, circumscribed, and continuous region bordering the third ventricle and ventral to the stria medullaris thalami. Additional anatomical exclusions to this definition included the centromedial and reuniens nuclei of the thalamus, which were both identified ventral to the PVT in coronal planes reaching from the mammillothalamic tract to the anterior extent of the red nucleus (Figure 4B). In the coronal plane, the PVT was widest anterior to the mammillothalamic tract (Figure 4A–C), narrowed as it extended posteriorly bordering the mediodorsal nucleus and the third ventricle (Figure 4D,E), and widened again at its posterior end (Figure 4G,H). Posterior to the interthalamic adhesion and ventral to the habenula, calretinin staining for cell bodies became markedly sparse and revealed predominantly calretinin‐stained fibers extending dorsolateral to the third ventricle, in what is often referred to as the “periventricular area” (Figure 4I,J). Given the distinct change in calretinin signal type in this region, we determined that these fibers were outside of the PVT.

### 3.2 Alignment of Histologically‐Defined PVT With MRI Images

The calretinin‐ and neuroanatomy‐defined PVT region as defined above was traced on the highest resolution MRI available for the post‐mortem excised thalamus block at 9.4 T (Figure 2A–D). We noted a slight skew to the coronal MRI plane, and therefore the tissue was sliced at a similar skew to assure alignment of the histological sections to the MRI plane (Figure 2A,B). Neighboring sections were used to confirm alignment of the histological plane to the MRI plane, and the PVT was traced onto the MRI using landmarks visible across modalities such as the stria medullaris thalamica (Figure 2C,D). Every histological section comprising the length of the PVT was confidently aligned to the MRI plane prior to tracing onto the MRI slabs and producing an individualized segmentation. The resulting three‐dimensional shape of the bilateral PVT was symmetrical, with a contour that closely matched the tissue‐based observations described above. Specifically, both left and right PVT were relatively wide anteriorly, narrowed along the extent of the mediodorsal thalamus, and exhibited a slight ventral dip (Figure 2E). The initial histologically grounded volume was approximately 63 mm^3^, encompassing 3148 voxels at 0.2 mm resolution. Following smoothing and registration to standard space at a lower, 0.5 mm voxel resolution, the new segmentation contained 1583 voxels (~198 mm^3^). In standard space, a critical comparison between this histologically grounded mask and the previously published Morel atlas‐based mask revealed a substantial degree of overlap in the anterior region, but a limited global overlap (DSC = 0.37). The histologically grounded mask also featured an additional posterior extension not present in the Morel atlas‐derived mask, which appears to contribute to the majority of non‐overlapping voxels (Figure 2F).

### 3.3 Functional Connectivity of the Histologically Validated Human PVT Mask

**FIGURE 5:**
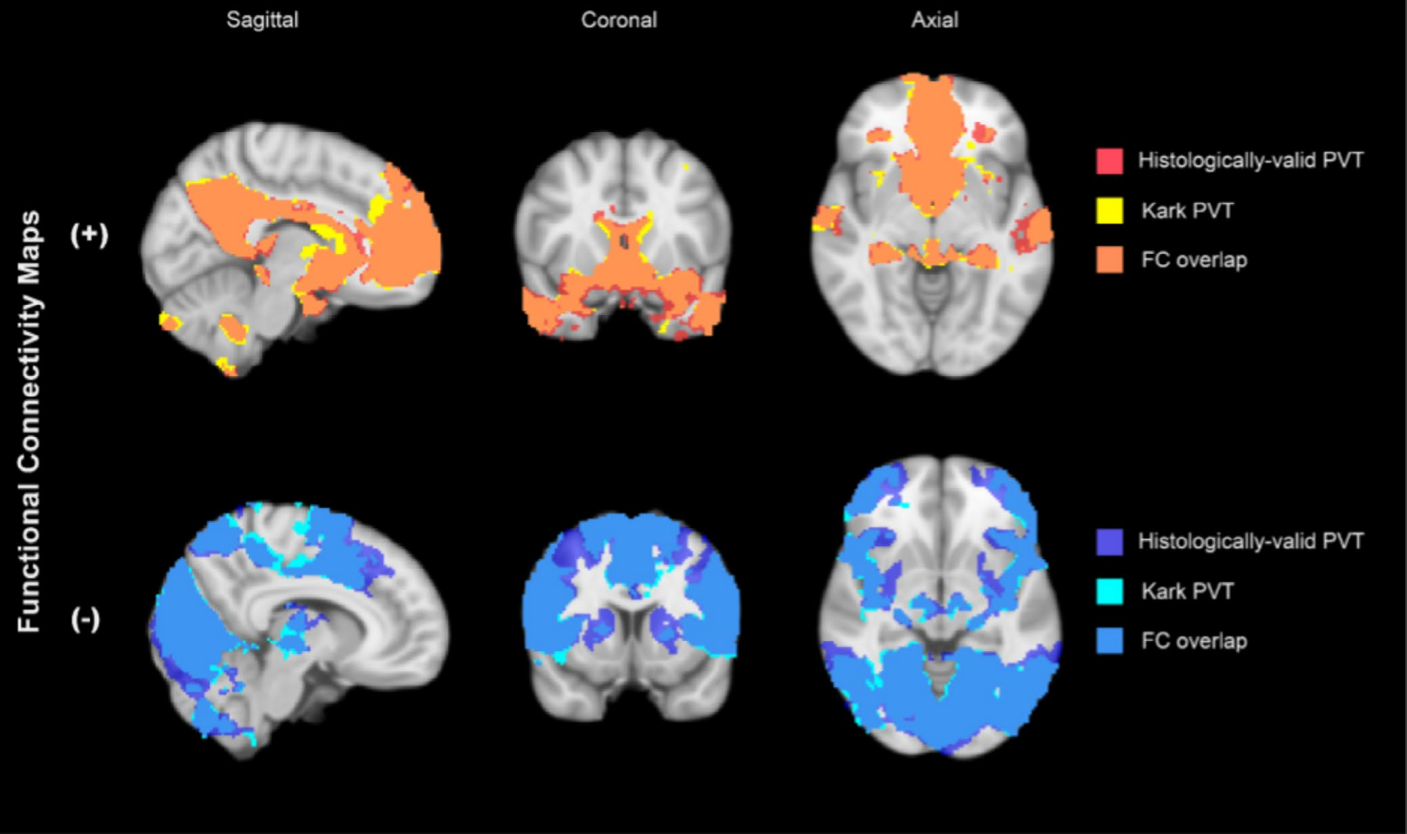
Functional connectivity maps are highly similar between the histology‐based mask and the PVT mask from Kark et al. 2021. Functional connectivity (FC) of the PVT was calculated by a seed‐to‐voxel analysis across all voxels of the brain. FC maps were generated following threshold‐free cluster enhancement (pTFCE‐FWE < 0.05) selection of significant voxels, and the maps from each analysis were divided by the sign (+/−) of the functional connectivity measures. Maps were then compared by calculating the Dice‐Sørensen coefficients (DSC) and coefficients of determination (*R* ^2^) to quantify similarity between maps. The results suggest highly similar patterns of PVT functional connectivity despite the difference in the PVT mask employed (Kark et al. 2021 segmentation vs. histologically valid segmentation presented in this study; positive FC maps: DSC = 0.854, *R* ^2^ = 0.851; negative FC maps: DSC = 0.848, *R* ^2^ = 0.857).

To assess the practical implications of the current and previous PVT masks in the context of in vivo human fMRI, we compared functional connectivity (FC) maps generated using the histologically grounded mask with the FC maps previously published using the Morel atlas. Overall, the two masks produced highly congruent whole‐brain connectivity patterns (Figure 5) with strong agreement in both positive FC maps (DSC = 0.854, *R* ^2^ = 0.851) and negative FC maps (DSC = 0.848, *R* ^2^ = 0.857). Importantly, as observed in prior studies using Morel atlas‐based masks, the functional connectivity of the human PVT remained consistent with findings from rodent studies. Regardless of the mask used, the human PVT exhibited strong connectivity with key nodes of both the reward and default networks, reinforcing cross‐species convergence in its functional organization.

## 4 Discussion

The key contributions of this study address challenges related to the translation across tissue‐based and MRI‐based definitions of the PVT. First, by integrating neuroanatomical landmarks with cell‐type‐specific immunohistochemistry, informed by modern atlases, we delineate the three‐dimensional boundaries of the human PVT with greater specificity than previously available. Second, we reanalyzed MRI‐based in vivo functional connectivity using this new mask and found it to be highly consistent with our previously published findings, thereby validating both the new tissue‐grounded masks and existing approaches as useful tools for studying this critical, yet understudied brain region. Finally, we provide the human neuroimaging community with the first segmentation of the human PVT derived directly from postmortem tissue and transformed into a commonly used MNI152 template space, allowing for increased interpretation between cellular characteristics and large‐scale in vivo brain networks.

### 4.1 Integrating Tissue‐Based and MRI‐Based Approaches Aids the Study of the Human PVT

Calretinin staining was used to distinguish the PVT from neighboring nuclei in postmortem tissue sections. Calretinin is an established marker of PVT across species. Unlike several markers identified for spatially segregated PVT cell types in mice, calretinin is expressed in a large proportion of PVT cells throughout the entire nucleus (Gao et al. 2023; Mátyás et al. 2018; Hua et al. 2018; Hsu et al. 2014; Viena et al. 2021). In humans, calretinin has been reported to express most densely in the PVT compared to surrounding nuclei, and to be expressed highly throughout the nucleus (Uroz et al. 2004; Sárkány et al. 2024). However, while calretinin is abundant in the PVT, it lacks specificity relative to the thalamic paratenial and reuniens nuclei, as well as the centromedial nucleus and the habenula. Nonetheless, these neighboring nuclei can be readily distinguished in coronal human tissue sections based on their proximity to the ventricular edge and position along the dorsoventral axis. We therefore delineated the PVT from the calretinin‐negative mediodorsal thalamic nucleus using calretinin, and from other calretinin‐positive nuclei using anatomical landmarks to produce a ground‐truth postmortem tissue definition of the human PVT.

In contrast to postmortem microscopy, the boundaries of small thalamic structures such as the PVT, paratenial nucleus, and reuniens nucleus are not easily distinguishable on standard‐resolution structural MRI. This limitation to precise localization necessitates the multimodal approach used here, while also requiring anatomical landmarks for cross‐modal integration. To ground our translation, we relied on myelinated structures that are clearly identifiable on both sectioned tissue and MRI, including the mammillothalamic tract and the anterior and habenular commissures. Based on our observations, these structures serve as reliable landmarks for anchoring masks of the PVT and other small midline thalamic nuclei within the neuroanatomy visible in MRI space (MNI152). Thus, future efforts to individualize MRI‐based segmentations of small, variable midline thalamic nuclei may benefit from using these landmarks as grounding points for transformation to standard space.

We observed substantial volumetric differences in the PVT when transforming from native space at ultra high resolution (0.2 mm isotropic at 7 T) to template space at high resolution (0.5 mm isotropic at 3 T). While this can largely be attributed to the spatial smoothing and quantization inherent in registration and down‐sampling processes, it also highlights challenges associated with translating small laminar structures to relatively low resolution spaces like those used in fMRI analysis. MRI‐based technologies continuously drift towards higher resolution acquisition, but at current resolutions this inflation is likely to remain, particularly when used for fMRI. A single, modern fMRI voxel is roughly 2 mm in width, wider than the widest coronal point of the PVT, suggesting that the partial sampling of surrounding nuclei is likely. This concern can be addressed in functional connectivity studies by regressing out the BOLD signal of voxels near PVT boundaries, as done previously by our group (Kark et al. 2021). We suggest that those investigating either the histological or functional human PVT should remain conscious of the atlases used to define the PVT in relation to the technology they implement.

As future work to study the PVT with either post‐mortem ex vivo or fMRI in vivo methodologies continues, our work provides a shared ground that could prove essential to comparative or correlative efforts across modalities. Specifically, appropriate comparison across these technologies allows consideration of the in vivo‐derived functional significance of any ex vivo‐derived cellular variations across the anterior–posterior axis of the human PVT. For example, important future work should evaluate the density and extent of functionally relevant markers such as orexinergic innervation across the human PVT as done previously in macaques (Hsu and Price 2009) and rodents (Kirouac et al. 2005). Such efforts could produce refined or sub‐nuclear parcellations of the PVT based on molecular level signaling testable in vivo by PET or other advanced imaging methods (Distler et al. 2024). We anticipate that recent advances in post‐mortem oriented technologies such as spatial transcriptomics are likely to dramatically improve our knowledge of small, variable human brain structures including the midline thalamus by allowing groups to generate high‐dimensional data with unprecedented anatomical precision. As this post‐mortem body of data grows, we believe that studies such as ours will serve as an essential bridge between functional, in vivo phenomena and the underlying cellular encoding processes.

### 4.2 Tissue‐Based Definitions of the PVT Extend Posterior Compared to Existing Available Atlases

No clear distinction between the anterior and posterior human PVT was observed in this study. Notably, this is in contrast to reports across species of an anterior and posterior division to the PVT. Specifically, rodent studies indicate functional and cellular differences across the anterior–posterior axis, with the anterior PVT associated with arousal and reward behaviors and the posterior PVT involved in stress and fear responses. Selective activity along this axis has been reported during motivated behavior (Beas et al. 2024) and as a result of early‐life stress (Kooiker et al. 2023). In humans, an anatomical division characterized by a thin laminar strip connecting the anterior and posterior PVT was recognized long before functional or cellular distinctions were characterized in any species. In terms of calretinin expression, we found no clear distinction between the anterior and posterior human PVT. However, the mid‐PVT narrowing we report is consistent with previous work dividing the human PVT into anterior and posterior segments and could represent a natural point of division. Toward the posterior end, the PVT gradually widens, with decreasing cell density that transitions into fiber tracts in the periventricular area (Dewulf 1971; Mai et al. 2004; Van Buren and Borke 1973). Thus, we propose that these fiber tracts are not within the PVT but instead constitute the posterior boundary. When comparing our histologically guided definition of the PVT to existing atlases in MRI space, we observed that this posterior extension is largely not captured by such atlases and represents an important area of future investigation.

Diverse perspectives exist regarding the extent and boundaries of the human posterior PVT. An additional region, confusingly referred to as the “periventricular area of the thalamus” (PeVa), is sometimes included as the posterior end of the PVT (Ding et al. 2016); in this region, we found calretinin‐positive fibers, but very few cell bodies, supporting the conclusion that this “PeVa” region is not part of the paraventricular *nucleus* of the thalamus. Notably, the terminology “paraventricular thalamus” is sometimes also used to describe the midline nuclei bordering the ventricle in the human; this likely contributes to confusion regarding the PVT and its borders, and we hope this work serves as clarification of the entity that is the human paraventricular *nucleus* of the thalamus, referred to here as the PVT.

### 4.3 Functional Connectivity Maps With and Without the Posterior End of the PVT Are Highly Similar

We present a new anatomically and histologically informed segmentation of the human PVT for use in in vivo human functional connectivity studies. Because this mask extends posteriorly relative to previous definitions, we compared functional connectivity maps derived from the new segmentation to those from our prior mask based on existing atlases. Notably, the inclusion of the posterior segment appeared to have minimal impact on the detection of functionally connected nodes using high spatial resolution fMRI data from the 7 T HCP Project. This unexpected finding may be due to: (1) the relatively large point‐spread function of fMRI combined with its limited spatial resolution reduces the detection of fine functional differences across the PVT axis, and/or (2) the larger volume of the anterior core of the PVT in both masks compared to the modest posterior addition in the new PVT mask, which carries greater weight in the average BOLD signal utilized in the functional connectivity map calculation for both masks. The high degree of overlap in functional connectivity between the two masks, along with their agreement with rodent connectivity patterns, supports the notion that human and rodent PVT networks target analogous regions. More specifically, analogous findings have been described in previous work by our group (Kark et al. 2021; Leonard et al. 2024), but notably, functional connectivity has been evaluated in rodents using immediate‐early genes (cFos) as early as the late 1990s and described in a recent editorial collection on the functional connections of PVT in rodents (Bhatnagar and Kirouac 2021). Given that the new mask spans both anterior and posterior portions of the PVT, and the previous version included primarily anterior PVT, our functional connectivity findings are consistent with the absence of histological differences on the posterior–anterior axis as revealed by calretinin staining. However, this result may be alternatively explained by the relatively large point‐spread function in fMRI, combined with the spatial extent and vascularization of the PVT, which may obscure fine‐grained functional distinctions (Fracasso et al. 2021). This study confirms the validity of prior work through the results of similar functional connectivity maps and provides a new resource to the field by integrating histological information into the PVT mask, and gives the foundation for future studies to further subdivide the PVT. More specifically, future studies leveraging higher‐resolution imaging and targeted functional analyses may reveal anterior–posterior specialization within the human PVT through a combination of statistical inference and data‐driven methods to parcellate the border.

Future studies leveraging higher‐resolution imaging and targeted functional analyses may reveal anterior–posterior specialization within the human PVT, with relevance for both normative and clinical populations. Additional evidence provided by histological studies for anterior and posterior PVT masks could open the field to probing convergence between rodent and human PVT function in each of these subregions. Rodent research demonstrates differential engagement of the anterior and posterior PVT in fear, stress, and reward (Beas et al. 2024; Gao et al. 2023, 2020; Kooiker et al. 2023), but the importance of the antero‐posterior distinction in the human PVT remains unknown. To address this, future human studies could combine cognitive tasks that elicit stress or reward responses and probe fear learning while evaluating posterior PVT engagement by specific task variables in fMRI study paradigms. Advances in understanding the functional role of the posterior PVT could be derived from measures of stress, such as heart rate variability, and enriched with varied neuroimaging techniques, such as functional connectivity to the amygdala or other related fear/stress regions, and structural imaging to quantify PVT volume or diffusion tractography metrics to evaluate communication between regions. Integrating this information uncovers what contribution the posterior PVT might have in clinical states, such as post‐traumatic stress disorder (PTSD), anxiety, withdrawal states, and general avoidance behavior computations.

While the current study was designed to address challenges in interpreting between studies performed at the cellular and meso/macroscopic scale, limitations remain—particularly regarding interindividual variability in borders not easily observed at current standard MRI resolutions. To help address this, we compared our tissue‐based segmentation, derived from a single individual, with existing prints of both individual and averaged post‐mortem thalamic parcellations. Based on qualitative comparisons, our donor‐derived PVT aligns with that of five postmortem cases used in the construction of the NextBrain atlas (Casamitjana et al. 2024), as well as with other established atlases (Mai et al. 2004; Toncray and Krieg 1946). Additionally, our mask shares morphological features with a diffusion‐based midline thalamus atlas generated by probabilistic tractography, though notably this atlas contains more subnuclei beyond the PVT (Reeders et al. 2023). Future diffusion‐based research could benefit from the findings of this study by integrating the presented, histologically validated PVT mask as a region of interest in tractography analyses or updating parcellation boundaries provided this new histological information. Specifically, we found that our PVT parcellation and others always begin the PVT slightly anterior to the mammillothalamic tract, PVT thins into a laminar strip as it proceeds posteriorly, and that the posterior edge of the PVT is at the level of the habenulae. Notably, some atlases show a slightly wider PVT anteriorly, and a slightly narrower PVT posteriorly as compared to our individual; these variations are on the scale of less than half a millimeter and may represent natural individual variation. The general agreement of our individual PVT with over 30 cumulative other reported cases of individual thalamic parcellations supports the accuracy of our delineation and suggests that our donor does not represent a gross anatomical outlier.

Nonetheless, substantial variation does exist in the human midline thalamus, including differences as obvious as the presence or absence of the interthalamic adhesion (massa intermedia) and gross branching of vascularization affecting the PVT region (Castaigne et al. 1981; Damle et al. 2017; Percheron 1973; Şahin et al. 2023; Vidal et al. 2024; Kochanski et al. 2018). Across the 25 cases averaged by Van Buren and Borke (1973), variation in the reported boundary for the mammillothalamic tract exceeded 5 mm, which would span multiple voxels at standard MRI resolutions. Given this variability, it is plausible that the averaged, anteriorly biased PVT present in the Morel and Krauth atlases represents a consistent “core” of the human PVT. Our findings extend this notion by demonstrating that functional connectivity derived from both our tissue‐based and the atlas‐based masks exhibits substantial overlap, supporting their shared utility in characterizing PVT.

Recent progress leveraging high‐resolution fMRI data has revealed brain‐wide functional connectivity patterns of the human PVT (Kark et al. 2021). The current study builds on this work by incorporating a histologically grounded definition of the human PVT. This newly developed mask is now publicly available in MNI152 space and in formats compatible with major neuroimaging toolkits (FSL, FreeSurfer, CIVET) via our GitHub repository (https://github.com/jerodras/pvt_mri_atlas_postm), ensuring broad accessibility for neuroimaging research. While the observations made here suggest that masks currently in popular use (i.e., Krauth) remain suitable for use in standard MR image processing pipelines and may reflect a “core” region of the PVT likely to be present across individuals, this newly available mask may provide a more comprehensive PVT including an additional posterior region. At present, we encourage use of the new histology‐informed mask for most human PVT fMRI studies, while noting that anterior‐focused analyses or continuity with earlier studies may justify use of the masks currently in popular use. The use of this new mask may provide further validation and new insights into the function of the human PVT in health and disease.

## 5 Conclusion

In summary, the current work provides the neuroimaging community with a valuable new resource: the first critical comparison between an anatomically and cell‐type informed segmentation of the human PVT and an average‐based mask of the PVT. It reconciles differences in extant atlases and affirms the validity of prior masks and their utility in analyses of PVT functional connectivity. We anticipate that this resource will become a useful tool to support future research into the structure and function of the human PVT, an important yet understudied brain region.

## Supporting information


**Data S1:** Supporting Information.

## Data Availability

The data that support the findings of this study are openly available in Human Connectome Project at https://www.humanconnectome.org.
