# Supplementary material for: A Combined Neuroanatomy, Ex Vivo Imaging, and Immunohistochemistry Defined MRI Mask for the Human Paraventricular Nucleus of the Thalamus
Source: Hum Brain Mapp. 2025 Sep 25;46(14):e70366. doi: 10.1002/hbm.70366 (PMC12462541; doi:10.1002/hbm.70366)
Supplement: Supplementary file 1 — Data S1: Supporting Information. [file HBM-46-e70366-s001.docx]

***Supplemental Analysis: Individual Standing in a Normative Sample***

To empirically contextualize the donor’s thalamic anatomy within a representative population, we analyzed FreeSurfer outputs from the Human Connectome Project Aging (HCP-A) cohort. Whole-thalamus volumes from the postmortem donor MRI were compared to distributions from the HCP-A sample (N=724). Thalamic subnuclei parcellation(1) was also performed on the donor MRI (Supplementary Figure S1, bottom middle panel) and on a subsample of 100 age-matched HCP-A participants (subsampled to reduce computational demands). Normative distributions adjusted for age, age-squared, sex, and brain volume (with the exception of brain volume itself) in the HCP-A cohort were used to generate z-scores for the donor’s whole brain, whole thalamus, and medial subnuclei volumes.

Several caveats to this analysis should be considered. First, postmortem tissue contrast differs from in vivo imaging. To address this, we used FreeSurfer’s synthSR(2) tool to generate synthetic T1-weighted contrast from the postmortem MRI (Supplementary Figure S1, top middle panel) prior to FreeSurfer processing. Second, the donor brain was fixed in 10% formalin, which likely caused some overall shrinkage. However, periventricular regions may also expand during early fixation, partially offsetting shrinkage(3). Third, subnuclei volumes are small and inherently more susceptible to noise, especially in single-subject analyses.

Overall, the donor’s whole brain, whole thalamus, and subnuclei volumes were within expected ranges for the HCP-A sample (Supplementary Figure S1, right panel). Whole-brain volume was consistent with moderate fixation-related shrinkage (z = –1.6, ~5th percentile, age- and sex-adjusted), whereas whole-thalamus volume was close to the population mean (z = +0.2, ~57th percentile, age- and sex-adjusted). The mediodorsal nucleus (MDm; encompassing much of the human paraventricular thalamus) was also within the normative range (z = +0.8, ~78th percentile). These findings support the conclusion that the donor’s global and relative thalamic volumes are broadly representative of the population.


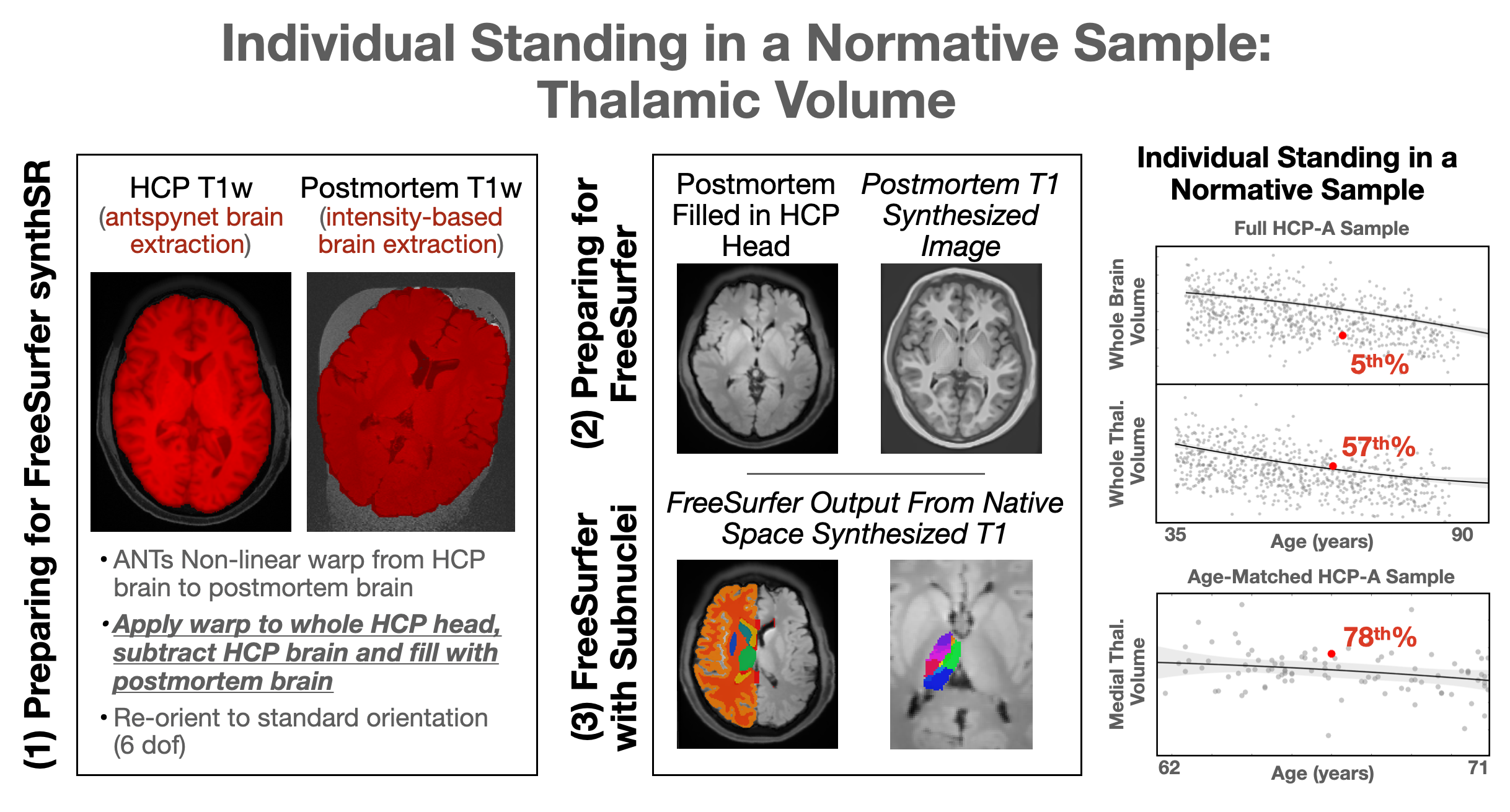


**Supplemental Figure S1: Individual Standing in a Normative Sample.** (Left panel) Postmortem MR image was prepared for FreeSurfer’s synthSR by replacing a random HCP-A participant brain with postmortem brain to simulate a whole head. (Middle panel) SynthSR was used to simulate T1-weighted contrast and perform FreeSurfer recon-all and subnuclei segmentation (medial thalamus volume used for percentile scoring is shown in bright green). (Right panel) Scatter plot depicting normative models used to percentile score the postmortem volumes (red).

| 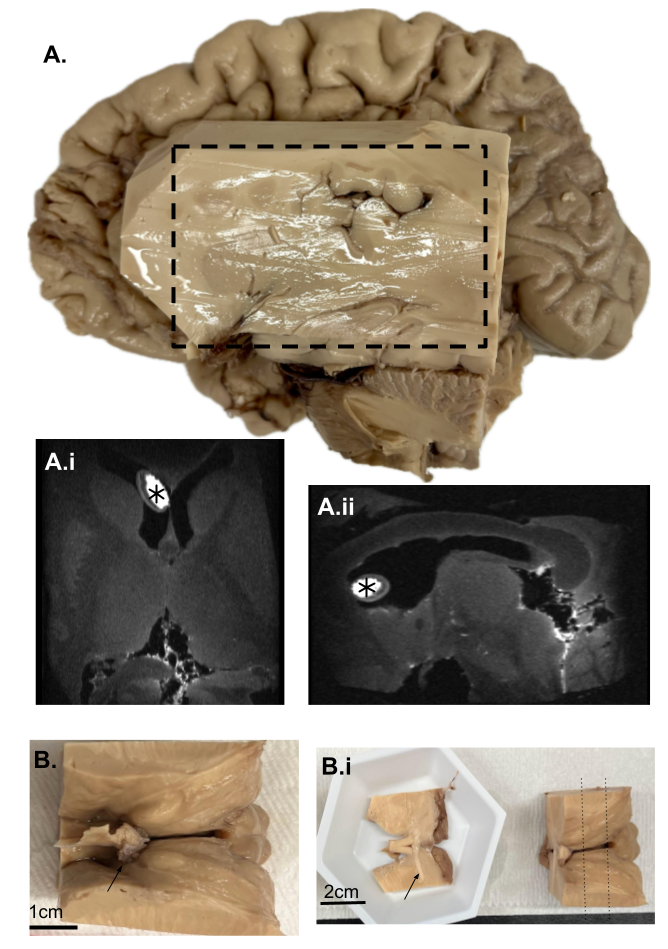  **Supplemental Figure S2: Tissue dissections.** (A) Cortex was dissected away from each side, leaving the ventricular space intact excluding incisions in the anterior and posterior extent to allow release of air and filling by fluorinert for optimal imaging quality. The brain halfway through this dissection is shown, with the left ventricle exposed. Following dissection, transverse (i) and sagittal (ii) MRI images of the thalamic block show the extent of the bilateral dissection as well as placement of vitamin E tablet for orientation (asterisk). Note that this dissection left the thalamus and adjacent ventricular system untouched for subsequent non-linear registration to the subject’s whole brain post-mortem MRI (collected prior to the dissection).Transverse (i) and sagittal (ii) MRI planes display the extent of the tissue block dissection indicated on A with a dotted line. (B) Following MRI acquisition, the ventricle was dissected away to reveal the bilateral thalamus and facilitate tissue processing. Beginning at the anterior commissure (arrow), 1.5 cm slabs (B.i) were cut, placed in trays, embedded in OCT, and snap-frozen for cryosectioning. |
| --- |


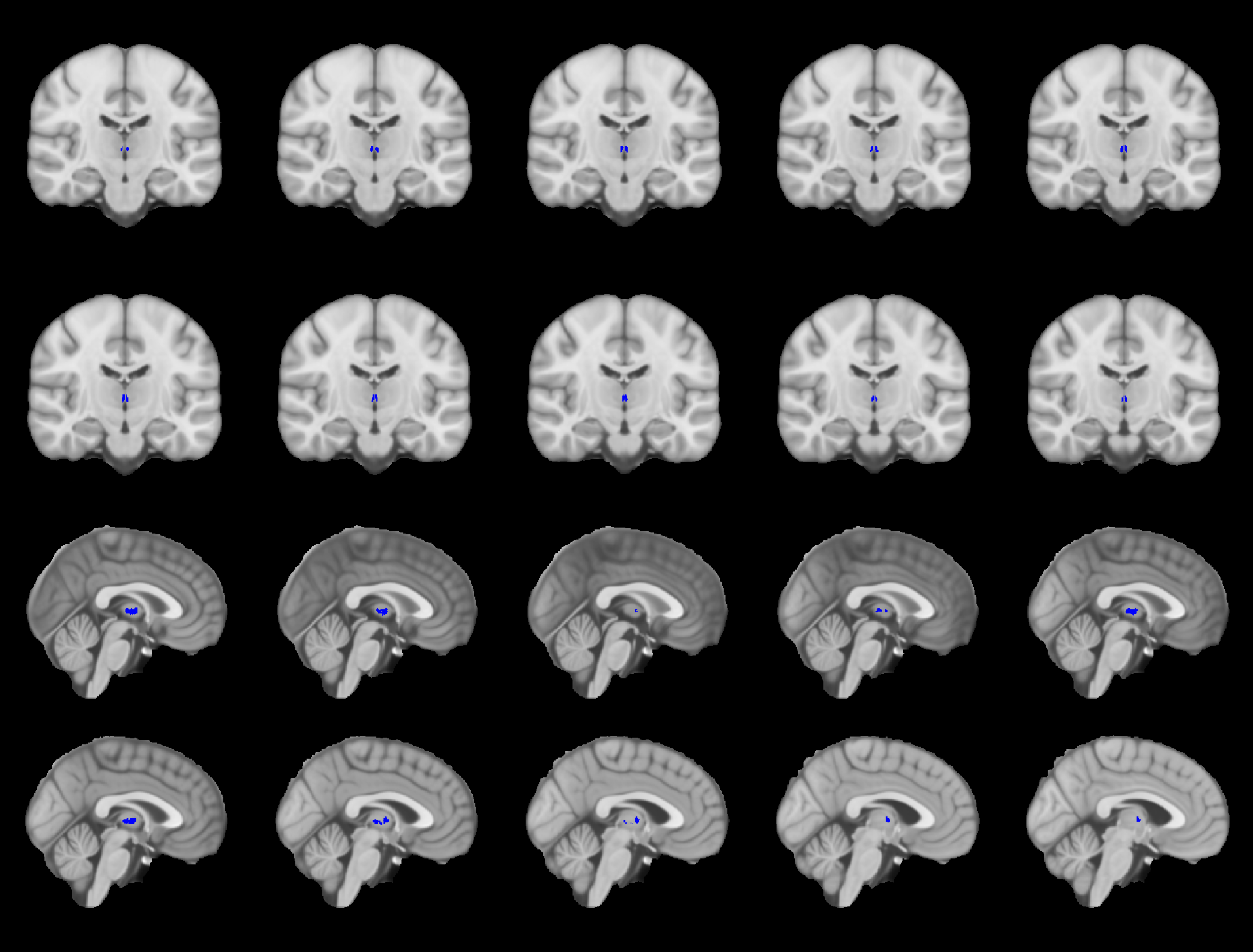


**Supplementary Figure S3: Additional views of final PVT mask.** Coronal and sagittal images are displayed of the final PVT mask (blue) at 0.5 mm^3^ voxel size on a MNI152 standard brain of the same resolution.

**Supplementary References**

1. Iglesias JE, Insausti R, Lerma-Usabiaga G, Bocchetta M, Van Leemput K, Greve DN, et al. A probabilistic atlas of the human thalamic nuclei combining ex vivo MRI and histology. Neuroimage. 2018;183:314-26.

2. Iglesias JE, Billot B, Balbastre Y, Magdamo C, Arnold SE, Das S, et al. SynthSR: A public AI tool to turn heterogeneous clinical brain scans into high-resolution T1-weighted images for 3D morphometry. Sci Adv. 2023;9(5):eadd3607.

3. Schulz G, Crooijmans HJ, Germann M, Scheffler K, Muller-Gerbl M, Muller B. Three-dimensional strain fields in human brain resulting from formalin fixation. J Neurosci Methods. 2011;202(1):17-27.
